# High psychological stress levels related to delivery can increase the occurrence of postpartum mental disorders

**DOI:** 10.3389/fpsyt.2023.1273647

**Published:** 2023-12-20

**Authors:** Ruixue Sun, Mingzhe Zhao, Liangkun Ma, Yanping Duan, Jing Wei

**Affiliations:** ^1^Department of Psychological Medicine, Peking Union Medical College Hospital, Chinese Academy of Medical Sciences and Peking Union Medical College, Beijing, China; ^2^Department of Gynecology and Obstetrics, Peking Union Medical College Hospital, Chinese Academy of Medical Sciences and Peking Union Medical College, Beijing, China

**Keywords:** postpartum, psychological stress, mental disorders, pregnancy, parturient

## Abstract

**Objective:**

The study sought to explore the relationship between high psychological stress levels related to delivery and postpartum mental disorders.

**Methods:**

A total of 284 parturients were included in the study from July 2021 to January 2022. The stress level at 1 month postpartum was assessed by the Impact of Event Scale-Revised (IES-R). Parturients with an IES-R score ≤ 9 were included in the low psychological stress level group, and those with an IES-R score > 9 were included in the high psychological stress level group. The Edinburgh Postnatal Depression Scale (EPDS), Union Physio-Psycho-Social Assessment Questionnaire (UPPSAQ-70), Symptom Checklist-90 (SCL-90) and Mini-International Neuropsychiatric Interview (M.I.N.I.) were conducted at 42 ± 7 days postpartum to assess the mental health of parturients.

The parturients’ mental health after birth was assessed by the EPDS, UPPSAQ-70, and SCL-90. Semi-structured diagnostic interviews were conducted at 42 ± 7 days postpartum by using the M.I.N.I.

**Results:**

The incidence rate of postpartum mental disorders was 20.42% (58/284), the incidence rates of postpartum depression, anxiety disorders, obsessive-compulsive disorder and posttraumatic stress disorder were 17.96% (51/284), 11.97% (34/284), 4.58% (13/284) and 1.41% (4/284), respectively, and the comorbidity rate was 58.62% (34/58). A history of mental disorders and pregnancy complications were risk factors for postpartum depression (*p* = 0.028, *p* = 0.040, respectively); a history of mental disorders, a lack of physical exercise, partner violence and pregnancy complications were risk factors for postpartum anxiety disorders (*p* = 0.003, *p* = 0.007, *p* = 0.031, *p* = 0.048, respectively); and the delivery of female infants was a risk factor for postpartum obsessive-compulsive disorder (*p* = 0.022).

The risk of postpartum depression, anxiety disorders and obsessive-compulsive disorder was 9.125 times (95% CI = 3.900 ~ 21.349, *p* < 0.01), 7.310 times (95% CI = 2.588 ~ 20.649, *p* < 0.01) and 6.259 times (95% CI = 1.347 ~ 29.093, p < 0.01) higher in postpartum women with high psychological stress levels related to delivery than in those with low psychological stress levels, respectively.

**Conclusion:**

The incidence of postpartum mental disorders is high and has a positive correlation with the level of psychological stress. This may lead to a new perspective of the effect of psychological stress on postpartum mental disorders and attract more attention to other mental disorders in addition to postpartum depression.

## 1 Introduction

Childbirth is one of the most influential factors inducing mental illness. All types of mental disorders may occur after childbirth (1). In addition to depression and anxiety disorders, postpartum mental disorders (PMD) also include acute stress disorder and posttraumatic stress disorder (PTSD), eating disorders, obsessive-compulsive disorder (OCD) and psychotic disorders. PMD will harm the physical and mental health of mothers and can even lead to suicide. For the offspring of mothers with these disorders, the risk of developmental delays, emotional regulation difficulties, poor cognitive development and social behavior problems in infancy (2, 3) and mental disorders in adolescence is increased (4). In addition, depression and anxiety symptoms are increased in fathers when mothers suffer from mental disorders (5), which has a devastating impact on family relations. Therefore, the ability to predict a person’s risk for developing PMD has clinically actionable implications. We can intervene early to reduce risk factors or conduct close psychological follow-up assessments on high-risk parturients.

A recent report from Simone explored a clinical risk index for common PMD. The independently associated variables include several sociodemographic and obstetric characteristics, such as mental health diagnosis history and medications during pregnancy, conception type and complications, lactation intention and so on (6). Ayer’s research suggests that childbirth can lead to PTSD, and the psychological stress reaction related to childbirth overlaps with various PMD, which lays the foundation for paying attention to maternal mental health from the perspective of stress reactions (7). Based on these thoughts, we try to explore the risk for PMD from the perspective of stress.

In this way, we explored the level of parturition-related physiological-psychosocial stress and its correlation with postpartum mental disorders through a diagnostic interview tool. We hope our study can provide a reference for the early detection and prevention of postpartum mental disorders.

## 2 Materials and methods

### 2.1 Research subject

The subjects of the study were pregnant women who filing and prenatal examination in obstetrics department of Peking Union Medical College Hospital from July 2021 to January 2022. The inclusion criteria were as follows: (1) 18–45-year-old females; (2) women who could read and write; and (3) women who signed the informed consent form. The exclusion criteria were as follows: (1) language, writing or reading disabilities; (2) abortion or stillbirth; and (3) questionnaires in which the number of completed items was less than 2/3 of the total items or the content was illogical.

### 2.2 Outcome measures

#### 2.2.1 Impact of event scale-revised (IES-R)

The IES-R, which is divided into 3 factors and has 22 items, is a self-evaluation scale for evaluating subjective responses to trauma. According to the severity of symptoms, a score of 0 to 4 points is given: 0 points for no impact, 1 point for a mild impact, 2 points for a moderate impact, 3 points for a severe impact, and 4 points for an extremely severe impact. The total score ranges from 0 to 88 points. A higher score predicts more serious symptoms. The internal consistency of IES-R is 1 in foreign studies and 0.89–0.96 in domestic studies. The split half reliability is 0.93 and.

3-week retest reliability is 0.51–0.94 (8). The IES-R has no strict cutoff value; in the study of nonobstetric trauma, it is generally considered that a score less than or equal to 9 indicates a low stress level (9).

#### 2.2.2 Edinburgh postnatal depression scale (EPDS)

The EPDS is a self-evaluation scale used for screening, auxiliary diagnosis and the evaluation of depression in perinatal women. There are 10 items in total. Each item is scored on a 4-point scale from 0 (never) to 3 (usually) according to the frequency of symptoms, and the total score ranges from 0 to 30 points. A higher score predicts a greater risk of depression. The internal consistency coefficient of the scale is 0.87, and the split half reliability is 0.88. Compared with the diagnostic criteria for depression used in the study, the sensitivity was 86% and the specificity was 78% (10). In China, a score of 9/10 points indicates the possibility of postpartum depression (the sensitivity is 0.82, and the specificity is 0.86) (10, 11); therefore, puerpera with an EPDS score ≥ 9 points were considered to have positive screening results in this study.

#### 2.2.3 Symptom checklist 90 (SCL-90)

The SCL-90, also known as the Self-Reporting Inventory, has 90 items, including items for wide range of psychiatric content such as feelings, emotions, thinking, consciousness, interpersonal relationships, diet and sleep. The SCL-90 is widely used in symptom measurement. Each item adopts a 5-point scoring system from 1–5 according to the severity of symptoms (1 for no, 2 for very light, 3 for medium, 4 for heavy, and 5 for serious). A total of 9 factors are included: somatization (12 items), obsessive-compulsive symptoms (10 items), interpersonal sensitivity (9 items), depression (13 items), anxiety (10 items), hostility (6 items), phobia (7 items), paranoia (6 items), and psychosis (10 items). The total SCL-90 score reflects the severity of disease. A higher score indicates more serious disease; factor scores reflect the characteristics of symptom groups. If the total score exceeds 160 points, the number of positive items exceeds 43, or any factor score exceeds 2 points, the screening is considered positive. According Derogatis’s report, the validity coefficients of each symptom in SCL-90 are 0.77–0.99, *p* < 0.01. Domestic and foreign comprehensive hospitals often use this scale to understand the mental symptoms of patients with physical diseases and believe that the results are satisfactory (12).

#### 2.2.4 Union physio-psycho-social assessment questionnaire (UPPSAQ-70)

The UPPSAQ-70 is a health screening self-assessment scale with three dimensions (physiological, psychological, social) that was independently developed and designed by the Department of Psychological Medicine of Beijing Union Medical College Hospital. The Chronbach’s α coefficient of each factor in UPPSAQ-70 is between 0.823 and 0.904. The Chronbach’s α coefficient of the full scale is 0.847, and there is a significant correlation between the two retests (*p* < 0.05) (13, 14). There are 70 items in the questionnaire, including 8 factors: emotions (9 items), sleep (8 items), anxiety and physical discomfort (18 items), pain (3 items), sexual function (5 items), happiness and satisfaction (9 items), hypochondriasis (12 items), and social interactions (6 items). Each item is scored according to the frequency or severity of symptoms on a 4-point scale from 0 to 3, with a total score ranging from 0 to 210 points. The higher the score is, the higher the symptom severity. If the total score of the equivalent table is ≥65 points or the average score of a single factor is ≥1 point, the screening result is considered positive.

#### 2.2.5 Mini-international neuropsychiatric interview (M.I.N.I.)

The M.I.N.I. is a short structured diagnostic tool for mental disorders. All items in the questionnaire are answered with yes/no, starting with screening questions and ending with diagnostic blocks, to check whether a patient meets the diagnostic criteria. This study used the Chinese version of M.I.N.I. 5.0, and the selected modules were as follows: A: major depressive episode (MDE); B: dysthymic disorder; C: suicidal tendency; D: (hypo) manic episode; E: panic disorder (PD); F: agoraphobia; G: social anxiety disorder; H: OCD; I: PTSD; J: alcohol abuse and dependence; K: nonalcohol psychoactive substance use disorder; L: psychotic disorder; M: anorexia nervosa; N: bulimia nervosa; and O: generalized anxiety disorder (GAD) (15). Studies in the United States, France, and Japan have found that the reliability Kappa values of M.I.N.I. are all greater than 0.75, with 70% of modules above 0.90 (12).

### 2.3 Study process

This study adopted a cross-sectional study design and a convenience sampling method to collect general information on mothers after childbirth in Peking Union Medical College Hospital. The information included the following age, nationality, occupation, medical insurance status, marital status, residence, education level, life status, family *per capita* income, height, weight, whether the pregnancy was planned, whether partner violence occurs, physical exercise (cardiorespiratory and/or resistance training, >30 min/day), drinking history (a pattern of pathological alcohol use or impairment in social or occupational functioning due to alcohol, and either tolerance or withdrawal), history of psychosocial diseases, partner history of mental and psychological diseases, whether the child was the first-born, mode of delivery, whether full-term delivery occurred, whether pregnancy complications (gestational diabetes and pregnancy-induced hypertension syndrome, hyperemesis gravidarum and placental abnormalities) occurred, and whether diabetes was diagnosed during pregnancy. The IES-R, EPDS, UPPSAQ-70 and SCL-90 were used to comprehensively evaluate and screen the conditions of the puerpera 42 ± 7 days after delivery. The time range of IES-R is 1 month postpartum, and the other scales evaluate the status of the past 1 week.

Parturients with an IES-R score ≤ 9 were included in the low psychological stress level group, and those with an IES-R score > 9 were included in the high psychological stress level group. Puerpera considered to have positive screening results were assessed and diagnosed with mental disorders by the M.I.N.I. 5.0. On the other hand, 30 % of the women with negative screening results were randomly selected for assessment with the M.I.N.I. The evaluation of this study was jointly completed by psychiatrists and graduate students qualified by the standardized training certificate in the department of psychiatry and mental health.

The research protocol, research process and informed consent form of this study were reviewed by the Ethics Review Committee of Peking Union Medical College Hospital with approval number ZS-2865. All subjects signed informed consent forms.

### 2.4 Statistical methods

This study used EpiData 3.1 to enter data into the database and IBM SPSS 25.0 to analyze the data. A 95% confidence interval was used in the test, and a two-sided t test was conducted. Differences were statistically significant at *p* < 0.05.

Descriptive statistics: the mean and standard deviation ( $\bar{X}$ ±s) were used to describe measurement data verified to conform to a normal distribution (comply with the principle of three standard deviations principles), and the frequency (n) and constituent ratio (%) were used to describe enumeration data.

Intergroup comparison: an independent sample t test was used for comparisons between measurement data groups that met the normal distribution, and the chi-square test or Fisher’s exact test was used for comparisons between counting data groups.

Correlation analysis: pearson correlation analysis was used for intergroup comparisons of measurement data.

Regression analysis: first, single-factor analysis was performed; then, statistically significant variables were incorporated into the multivariate binary logistic regression model to analyze the influencing factors. The unconditional logistic regression model was selected to correct for confounding factors, and the odds ratio (OR) and 95% confidence interval were calculated.

## 3 Results

### 3.1 Sociodemographic characteristics and obstetric data

A total of 337 parturients signed the informed consent form, of which 22 were not included in the study because the number of items completed in the scale was less than 2/3 of the total number of items. Eight parturients could not be contacted during the study, and 23 parturients refused to undergo the Concise International Neuropsychiatric Interview. Therefore, a total of 284 parturients were included in the study, and the response rate was 84.27%. The average age was 33.20 ± 3.62 years old, and most of the parturients were married (99.30%, 282/284), in-service (96.13%, 273/284), living with others (98.59%, 280/284), had a high education level (95.77%, 272/284) and had good economic status (85.56% had a family average monthly income of 8,000 yuan or above).

Combined with previous studies (12), the revised version of the impact scale with 284 maternal events used in this study was determined according to the distribution within the group. The median IES-R score was 9, which was defined as the cutoff value. Parturients with an IES-R score ≤ 9 were included in the low psychological stress level group, and those with an IES-R score > 9 were included in the high psychological stress level group. Comparing the sociodemographic characteristics of the high and low psychological stress level groups, there was no significant difference in age, nationality, occupation, marital status, living conditions, monthly income, drinking history, body mass index (BMI), or physical exercise level between the two groups (*p* > 0.05). The education level of the high psychological stress level group was higher than that of the low psychological stress level group. There was no significant difference in the composition between the two groups (Table 1).

**Table 1:** Sociodemographic characteristics.

Variable	Population (*n* = 284)	Low stress level group (*n* = 145)	High stress level group (*n* = 139)	*t*/*χ*^2^	*P*
Age (years)	33.20 ± 3.62	33.43 ± 3.81	32.96 ± 3.42	1.078	0.282
Nationality *n* (%)				1.271	0.260
Han	257 (90.49)	134 (92.41)	123 (88.49)		
Others	27 (9.51)	11 (7.59)	16 (11.51)		
Occupation *n* (%)				0.056	0.813
In service	273 (96.13)	139 (95.86)	134 (96.40)		
Unemployment	11 (3.87)	6 (4.14)	5 (3.60)		
Marital status *n* (%)				0.001	0.976
Married	282 (99.30)	144 (99.31)	138 (99.28)		
Other	2 (0.70)	1 (0.69)	1 (0.72)		
Education level *n* (%)				8.269	0.005
Above bachelor	272 (95.77)	134 (92.41)	138 (99.28)		
Under bachelor	12 (4.23)	11 (7.59)	1 (0.72)		
Living conditions *n* (%)				0.931	0.623
Not living alone	280 (98.59)	142 (97.93)	138 (99.28)		
Living alone	4 (1.41)	3 (2.07)	1 (0.72)		
Per monthly income *n* (%)				0.099	0.866
Above ¥8,000	243 (85.56)	125 (86.21)	118 (84.89)		
Under ¥8,000	41 (14.44)	20 (13.79)	21 (15.11)		
Drinking history				0.939	0.372
Yes	193 (67.9)	102 (70.34)	91 (65.47)		
No	91 (32.04)	43 (29.66)	48 (34.53)		
BMI (kg/m ^2^)	23.08 ± 2.77	23.21 ± 2.53	22.95 ± 3.01	0.494	0.622
Physical exercise *n* (%)				0.045	0.893
Yes	210 (73.94)	108 (74.48)	102 (73.38)		
No	74 (26.06)	37 (25.52)	37 (26.62)		

BMI, body mass index.

Among the 284 parturients included in this study, most delivered their first-born child (77.46%, 220/284), had a planned pregnancy (86.97%, 247/284), had a full-term delivery (89.08%, 253/284), and had a vaginal delivery (61.62%, 175/284), and the male-to-female ratio of newborns was balanced. The obstetric characteristics of the high and low stress level groups were compared. There was no significant difference between the two groups in terms of the mode of conception, whether the child was the first-born, whether the pregnancy was planned, newborn sex, whether the delivery occurred full-term, whether pregnancy complications and maternal complications occurred, and whether gestational diabetes mellitus (GDM) was diagnosed during pregnancy (*p* > 0.05) (Table 2).

**Table 2:** Obstetric data.

Factor	Population (*n* = 284)	Low stress level group (*n* = 145)	High stress level group (*n* = 139)	*χ*^2^	*P*
Mode of production *n* (%)				0.329	0.566
Delivery	175 (61.62)	87 (60.00)	88 (63.31)		
Cesarean section	109 (38.38)	58 (40.00)	51 (36.69)		
Primary *n* (%)				0.436	0.571
Yes	220 (77.46)	110 (75.86)	110 (79.14)		
No	64 (22.54)	35 (24.14)	29 (20.86)		
Planned pregnancy *n* (%)				0.001	1.000
Yes	247 (86.97)	126 (86.90)	121 (87.05)		
No	37 (13.03)	19 (13.10)	18 (12.95)		
Newborn sex *n* (%)				0.051	0.822
Male	147 (51.76)	76 (52.41)	71 (51.08)		
Female	137 (48.24)	69 (47.59)	68 (48.92)		
Term production *n* (%)				0.099	0.753
Yes	253 (89.08)	130 (89.66)	123 (88.49)		
No	31 (10.92)	15 (10.34)	16 (11.51)		
Pregnancy complications *n* (%)				0.101	0.753
Yes	48 (16.90)	21 (14.48)	27 (19.42)		
No	236 (83.10)	124 (85.52)	112 (80.58)		
Gestational diabetes mellitus *n* (%)				1.234	0.273
Yes	62 (21.83)	28 (19.31)	34 (24.46)		
No	222 (78.17)	117 (80.69)	105 (75.54)		

### 3.2 Prevalence and clinical characteristics of postpartum mental disorders

The incidence of postpartum mental disorders was 20.42% (58/284). Among the 284 parturients, 51 (17.96%) had a depressive episode, 2 of whom had suicidal tendencies, accounting for 3.92% of the sample. Five parturients had major depressive episodes with melancholic features, and 14 reported a history of depressive disorder before delivery; 4 (1.41%) were in a (mild) manic state; 3 had panic disorder (1.06%); 5 had phobias regarding places (1.76%); and 2 had social phobia (0.70%). There were 28 patients (9.86%) with generalized anxiety disorder (GAD). There are a total of 34 individuals diagnosed with anxiety disorders, with an incidence rate of 11.97%. Three patients reported a history of anxiety disorder before delivery, 13 reported OCD (4.58%), and 4 reported PTSD (1.41%). The comorbidity rate of mental disorders was 58.62% (34/58) (Table 3).

**Table 3:** Prevalence of postpartum mental disorders.

Mental disorders	Number of people (*n*)	Incidence (%)
Depressive episode	51	17.96
Suicidal tendencies	2	0.70
(mild) manic state	4	1.41
Anxiety disorder	34	11.97
Panic disorder	3	1.06
Place phobia	5	1.76
Social phobia	2	0.70
Generalized anxiety disorder	28	9.86
Obsessive compulsive disorder	13	4.58
Post-traumatic stress disorder	4	1.41
Mental disorders	58	20.42

### 3.3 Risk factors for postpartum mental disorders

The risk factors for postpartum mental disorders included age, nationality, occupation, medical insurance status, marital status, residence, education level, living condition, family *per capita* monthly income, BMI, whether the pregnancy was planned, whether partner violence occurred, physical exercise level, drinking history, history of mental illness, history of partner mental illness, whether the child was the first-born, mode of delivery, whether full-term delivery occurred, and whether pregnancy and maternal complications occurred. The univariate analysis of whether GDM diagnosed during pregnancy was a risk factor for postpartum mental disorders showed that compared with the healthy group, in the postpartum depression group, the distribution of physical exercise levels, history of mental and psychological diseases, premature birth, pregnancy complications and maternal complications were significantly different. There were significant differences in the distribution of partner violence (including physical violence, psychological violence, and sexual violence), physical exercise levels, history of mental and psychological disease, premature birth, pregnancy complications and maternal complications between the puerpera with postpartum anxiety disorders and the healthy group. There were significant differences in neonatal sex and pregnancy and maternal complications between the puerpera with postpartum OCD and the healthy group (Table 4).

**Table 4:** Univariate analysis of risk factors for postpartum mental disorders.

	Depression disorder Anxiety disorder Obsessive-compulsive disorder					
Factor	*χ*^2^/*t*	*P*	*χ*^2^/*t*	*P*	*χ*^2^/t	*P*
Age	0.607	0.545	1.050	0.294	1.224	0.222
Nation	0.949	0.247	0.021	0.591	0.052	0.644
Occupation	0.000	0.618	0.419	0.388	0.549	0.592
Medical insurance	0.066	0.633	0.036	0.595	0.344	0.718
Marital status	0.441	0.673	0.274	0.775	0.097	0.910
Residence	1.342	0.301	0.834	0.462	0.294	0.753
Educational level	2.742	0.088	1.704	0.210	0.601	0.563
Living conditions	0.888	0.451	0.552	0.599	0.195	0.828
Household *per capita* monthly income	0.359	0.549	0.233	0.798	0.502	0.700
BMI	1.263	0.209	0.804	0.423	0.496	0.621
Planned pregnancy	0.570	0.450	0.096	0.757	1.214	0.232
Partner violence	4.272	0.073	8.411	0.024	0.294	0.753
physical exercise	4.046	0.044	8.843	0.003	1.088	0.297
History of mental illness	4.780	0.045	10.484	0.008	0.405	0.437
History of partner mental illness	0.220	0.820	0.136	0.880	0.048	0.954
Primipara	0.851	0.356	2.567	0.129	1.719	0.310
Mode of production	0.595	0.441	0.538	0.463	0.334	0.772
Newborn sex	1.851	0.174	1.733	0.188	7.220	0.009
Term production	4.830	0.028	6.320	0.012	0.146	0.574
Pregnancy complications	5.536	0.019	7.879	0.005	3.888	0.049

BMI, body mass index.

The factors with significant differences in univariate analysis were included in multivariate binary logistic regression analysis. A history of mental illness, pregnancy complications and maternal complications were risk factors for postpartum depression. A history of mental illness, partner violence, pregnancy complications and maternal complications were risk factors for postpartum anxiety disorders, and physical exercise was a protective factor against postpartum anxiety disorders. Neonatal sex was associated with postpartum OCD (the incidence of postpartum OCD was higher in women who delivered female neonates). The risk of postpartum depression and anxiety disorders in puerperal women with a history of mental and psychological disease was 3.542 times and 6.734 times than that in puerperal women without a history of mental and psychological disease, respectively (Table 5).

**Table 5:** Multivariate binary logistic regression analysis of influencing factors of postpartum depression, anxiety disorder and OCD.

	Factor	*β*	Se	Wald *χ*^2^ value	*P*	Or
Postpartum depression	History of mental illness	1.265	0.574	4.848	0.028	3.542
	Pregnancy complications	0.778	0.378	4.237	0.040	2.176
Postpartum anxiety	History of mental illness	1.907	0.636	9.002	0.003	6.734
	physical exercise	1.115	0.413	7.292	0.007	0.328
	Partner violence	2.058	0.956	4.637	0.031	7.831
	Pregnancy complications	0.893	0.452	3.905	0.048	2.442
Postpartum OCD	Fetal sex	1.786	0.781	5.223	0.022	0.168

OCD, obsessive-compulsive disorder.

### 3.4 Relationship between high psychological stress levels related to childbirth and postpartum mental disorders

The incidence of postpartum mental disorders (including depression, generalized anxiety disorder, OCD, and PTSD) in the high psychological stress level group and the low psychological stress level group significantly differed (*p* < 0.05), and the incidence of mental disorders in the high psychological stress level group was higher (Table 6).

**Table 6:** Comparison of the incidence of mental disorders between the high psychological stress level group and the low psychological stress level group.

Mental disorders	Low stress level group (*n* = 145)	High stress level group (*n* = 139)	*χ*^2^	*P*
Depressive disorder *n* (%)	7 (4.83)	44 (31.65)	34.667	< 0.001
Anxiety disorder *n* (%)	5 (3.45)	29 (20.86)	20.424	< 0.001
Generalized anxiety disorder	4 (2.76)	24 (17.27)	*	< 0.001
Panic disorder	1 (0.69)	2 (1.44)	*	0.616
Place phobia	1 (0.69)	4 (2.88)	*	0.206
Social phobia	0	2 (1.44)	*	0.239
OCD *n* (%)	2 (1.38)	11 (7.91)	*	0.010
PTSD *n* (%)	0	4 (2.88)	*	0.040
Mental disorder *n* (%)	10 (6.90)	48 (34.53)	33.351	< 0.001

*Was using Fisher exact test.

OCD, obsessive-compulsive disorder.

According to the results of the M.I.N.I., the psychological stress levels of postpartum women with mental disorders and healthy women were compared, and there were significant differences between the two groups. Postpartum women with depression, social phobia, OCD, PTSD and anxiety disorders had significantly higher levels of psychological stress related to childbirth than healthy women (*p* < 0.05) (Table 7).

**Table 7:** Comparison of psychological stress levels between postpartum mental disorder group and normal group.

	Psychological stress level (^−^x ± s)			
Mental disorders			*T*	*P*
	Diseased group	Normal group		
Depressive disorder	25.02 ± 9.94	9.94 ± 9.47	9.800	< 0.001
(mild) mania	28.00 ± 4.24	12.54 ± 11.47	1.903	0.058
Panic disorder	14.67 ± 7.51	12.63 ± 11.55	0.305	0.761
Agoraphobia	15.60 ± 7.733	12.59 ± 11.56	0.578	0.564
Social phobia	33.00 ± 15.56	12.50 ± 11.38	2.535	0.012
OCD	24.15 ± 12.27	12.10 ± 11.20	3.777	< 0.001
Post-traumatic stress disorder	43.75 ± 0.50	12.20 ± 10.97	5.746	< 0.001
Generalized anxiety disorder	25.79 ± 12.87	11.21 ± 10.41	6.864	< 0.001
Mental disorders	24.02 ± 12.34	9.73 ± 9.27	9.736	0.003

OCD, obsessive-compulsive disorder.

The risk of depressive disorder, anxiety disorder and OCD were 9.125 times, 7.310 times and 6.259 times higher in the high psychological stress level group than in the low psychological stress level group, respectively (*p* < 0.05). A high psychological stress level related to childbirth had the greatest impact on the occurrence of postpartum depression, as shown in Table 8.

**Table 8:** Correlation between high psychological stress level in childbirth and postpartum mental disorder.

Variable	*M*odel 1			*M*odel 2		
	OR	95%CI	*P*	OR	95%CI	*P*
Postpartum mental disorder						
Low stress level	1.000			1.000		
High stress level	7.121	3.427 ~ 14.796	< 0.001	7.108	3.337 ~ 15.138	< 0.001
Postpartum depression						
Low stress level	1.000					
High stress level	9.131	3.945 ~ 21.135	< 0.001	9.125	3.900 ~ 21.346	< 0.001
Postpartum anxiety disorder						
Low stress level	1.000			1.000		
High stress level	7.382	2.767 ~ 19.696	< 0.001	7.310	2.588 ~ 20.649	< 0.001
Postpartum obsessive-compulsive disorder						
Low stress level	1.000			1.000		
High stress level	6.145	1.337 ~ 28.246	0.020	6.259	1.347 ~ 29.093	0.019

Model 1, unadjusted confounders; Model 2, adjusted for risk factors.

CI, confidence interval.

## 4 Discussion

Under acute stress, the sympathetic adrenal medullary system (SAM) and hypothalamic–pituitary axis (HPA) are highly activated, releasing catecholamines (mainly epinephrine) and glucocorticoids to accelerate glucose oxidation and glycolysis, promoting protein and fat breakdown, and ensuring normal organ function (16). However, when the stress level is too high, the HPA is hyperexcitable, and cortisol levels increase significantly. Studies have found that an increase in cortisol levels is related to the development of depression (17). Pregnancy and childbirth are stressful life events for women of childbearing age. Mental disorders are prone to occur due to substantial changes in physiology and the continuous stimulation caused by mental stress (18). Our study found that a variety of mental disorders occurred in the postpartum period, and comorbidities were very common. There was a significant positive correlation between the level of psychological stress related to delivery and postpartum mental disorders. This result may lead to a new perspective of the effect of psychological stress on postpartum mental disorders and attract more attention to other mental disorders in addition to postpartum depression.

### 4.1 Incidence of postpartum mental disorders

Postpartum depression was the most common mental disorder in the perinatal period. The incidence of depressive episodes in this study was 17.96%, which was comparable to Hahn Holbrook’s review of 291 studies worldwide (19). Maternal suicide is a serious adverse consequence of postpartum depression. In this study, 3.92% of postpartum women with depression had suicidal tendencies. Brockington et al. (20) found that 4.5% of 535 postpartum patients with depression were at risk of suicide. It is necessary to screen and evaluate the suicidal tendency of patients with postpartum depression. It is also important to note that 1.41% of postpartum women showed a (mild) manic state. Previous studies indicate that the first episode of postpartum depression may indicate potential bipolar disorder (21). At present, medical staff mostly focus on postpartum depression, and we should prevent missed diagnoses of bipolar disorder.

Postpartum anxiety is very common but is also misunderstood as a normal phenomenon for new mothers. In this study, the incidence of anxiety disorder in the postpartum period was 11.97%. A previous study of patients in nonpsychiatric outpatient departments of 9 general hospitals in three cities found that the detection rate of anxiety disorder was 7.6% (22), and the incidence of anxiety disorders in the postpartum period was relatively high. Panic disorder, place phobia, social phobia, and generalized anxiety disorder occur in the postpartum period and may coexist. It is very important to educate postpartum women and their families to understand anxiety disorders and seek help as soon as possible.

Similarly, compared with the general population, women are more likely to suffer from OCD after childbirth (23). In previous studies, the incidence of postpartum first-episode OCD was approximately 2.3–11% (24, 25). The incidence in this study was 4.58%. Both obsessive-compulsive thoughts and behaviors occurred and were related to newborns. In our interviews, some respondents had obsessive-compulsive symptoms, but the severity did not constitute OCD. This part of the population also deserves attention and early intervention to prevent the development of OCD.

Traumatic experiences during pregnancy and traumatic childbirth can cause PTSD. In this study, the incidence of postpartum PTSD was 1.41%. Foreign countries previously reported that the prevalence of postpartum PTSD in high-income countries was approximately 1–2%, while the prevalence in low-income countries was higher, at approximately 5–8% (26–28).

In this study, the incidence of mental disorders 42 ± 7 days after birth was 20.42%, and more than half of the patients had comorbidities. Previous studies have shown that postpartum depression is often comorbid with anxiety disorder (29), and PTSD is highly comorbid with depression (30). This study supports the above conclusions. The conditions of patients with comorbidities are complex and serious, which makes their diagnosis and treatment challenging, and these patients should be given more attention.

### 4.2 Influencing factors of postpartum mental disorders

In this study, there was a correlation between a history of previous mental illness and the occurrence of postpartum depression and anxiety disorders, which was consistent with the results of previous studies. A prospective study in Germany found that having a history of depression before pregnancy increases the risk of postpartum depressive symptoms (31). A literature review by Robertson suggested that anxiety or depression during pregnancy or a history of depression were the strongest predictors of postpartum depression (32). Women with a history of depressive disorder were 2.6 times more likely to have postpartum anxiety symptoms than women without a history of depression (33). Women with a history of mental illness face complex problems of disease recurrence and prognosis during pregnancy and the postpartum period, and their risk of mental disorders increases after childbirth. Medical staff should pay more attention to mothers with a history of mental and psychological diseases, remind them of possible changes in their condition, and pay close attention to changes in their symptoms.

Pregnancy complications and maternal complications are risk factors for postpartum depression. The results of a population-based mother-infant cohort in Crete, Greece, showed that women with pregnancy-induced hypertension and preeclampsia had more postpartum depressive symptoms (34), which was consistent with the findings of Blom et al. (35). Women with pregnancy complications and other complications are more prone to premature birth. Worrying about the health of herself and her newborn can make a mother more stressed and prone to emotional problems.

According to our results, regular exercise during pregnancy is a protective factor against postpartum anxiety disorder. Koltyn et al. (36) studied postpartum women and found that after 60 min of low-intensity aerobic exercise, maternal anxiety scores were significantly reduced, and emotions were significantly improved; Cram Ag and others also reached the same conclusion using the State–Trait Anxiety Inventory (STAI) to evaluate the emotional state of pregnant women after exercise. A large randomized controlled trial involving 189 pregnant women in Iran found that (37) lifestyle interventions, including physical exercise during pregnancy, can significantly reduce the postpartum anxiety score. In addition, there is sufficient evidence to prove that moderate and regular physical exercise can also prevent the occurrence of depressive disorder to a certain extent (38, 39). Moderate physical exercise during pregnancy is very beneficial.

In this study, domestic violence was a risk factor for postpartum anxiety disorders. Domestic violence committed by partners against women during pregnancy mainly includes physical violence, psychological violence (including verbal or emotional abuse), and sexual violence, of which psychological violence is the most common (40). A cross-sectional study of 373 pregnant women enrolled in the Nigerian primary health center conducted by Mapayi et al. (41) showed that women who experienced domestic violence were 10 times more likely to report anxiety than those the control group. A systematic review and meta-analysis of 67 papers showed that (42) domestic violence was significantly associated with high levels of postpartum anxiety, which was consistent with the findings of this study. Domestic violence is a global public health problem. In addition to the increase in postpartum anxiety, studies have also found that domestic violence increases the risk of adverse outcomes such as miscarriage, premature birth, fetal distress, and low birth weight (43). Perinatal nursing and health care professionals should pay attention to the identification of domestic violence, especially psychological violence, and provide timely support and assistance to pregnant and postpartum women. It may be of great significance to reduce postpartum anxiety.

At present, there are few studies on the risk factors for postpartum OCD. Studies that link neonatal sex with postpartum mental disorders mainly focus on postpartum depression, and no unified conclusion has been reached. Studies in Western countries have not found a significant association between neonatal sex and postpartum depression (39), but studies in low-income countries such as India and Nigeria have found a significant correlation between the birth of female infants and postpartum depression (44, 45). Xie et al.’s (46) research results in China showed that after adjusting for potential confounders, the risk of postpartum depression in women who gave birth to female infants was twice that of women who gave birth to male infants. The researchers speculated that this was related to the social and cultural background. Family members may have more negative reactions to the birth of female infants, so mothers may receive less family support. In this study, giving birth to girls may be associated with maternal postpartum OCD. In addition, an increasing number of studies have found that fetal sex can specifically affect the maternal immune and endocrine systems. For example, male fetal sex is related to the increase in maternal proinflammatory cytokines, and fetal sex can lead to changes in maternal reproductive hormone levels (47, 48). This provides the basis for the link between neonatal sex and postpartum mental disorders. Our study found that neonatal sex may be associated with the occurrence of postpartum OCD, which provides a new idea for the follow-up study of postpartum OCD.

### 4.3 Correlation between high psychological stress levels related to childbirth and postpartum mental disorders

Our study showed that the risk of postpartum depression in the high stress level group was 9.125 times higher than that in the low stress level group. This is consistent with the view of Soderquist et al. (49) that there is a correlation between postpartum acute stress and postpartum depression. Childbirth-related psychological stress and depression disorders have overlapping symptoms, such as a continuous inability to experience positive emotions, sleep disorders, and difficulty concentrating (50). From the perspective of high-risk groups, people with low self-evaluation, pessimism, sensitivity, avoidant coping styles, and a lack of social support are prone to higher stress when exposed to external stimuli, and these personality characteristics are also risk factors for depression. In terms of pathogenesis, previous studies have shown that stressful life events can change the epigenetic modification of genes, and women with postpartum depression have different methylation patterns of the hp1bp3 and ttc9b genes (51, 52). Stress stimuli can produce many free radicals. When the body’s antioxidant reduction capacity is exceeded, the body enters a state of oxidative stress. Similarly, patients with depression have oxidative stress damage in their brain and peripheral blood (53). Stress causes the HPA axis to be hyper functional, and the level of cortisol steroids increases continuously, which can lead to changes in the structure and function of brain tissue, thus causing depression. Psychological stress causes immune cell behavior disorders, which may increase cytokines in the brain to pathological levels, interfere with signal transmission, neurotransmitter synthesis, reuptake and release, and affect the emotional and cognitive functions of the nervous system (54).

In this study, the risk of postpartum anxiety in the high stress level group was also higher. Stress can lead to increased levels of norepinephrine (NE), which can stimulate thalamic α receptors, leading to increased alertness and inducing strong feelings of anxiety, fear, and anxiety-like behavior (16). Under high psychological stress, the activity of the limbic system, including the amygdala, anterior cingulate gyrus and prefrontal cortex, increases. Studies have found that the increase in volume and activity of these parts of the brain are positively correlated with the severity of symptoms of generalized anxiety disorder (55). Previous studies have also found that people with high anxiety traits are more susceptible to traumatic events, experience more symptoms and have higher stress levels after trauma, and these people are also at high risk for postpartum generalized anxiety disorder (3).

After adjusting for possible confounding factors, the risk of OCD in puerpera with high stress levels was 6.259 times higher than that in those with low stress levels. Research on psychological and behavioral responses to stress has found that people often have repeated ineffective and involuntary thoughts or actions when faced with strong stimuli or major setbacks (24). From the perspective of behavioral theory, patients may take certain actions to alleviate the anxiety caused by some situations. After patients believe that these compulsive or ritual behaviors can alleviate anxiety, they gradually increase compulsive behaviors through conditioned reflexes and even generalize these behaviors (25). For example, in clinical practice, a new mother may worry about her inability to take care of her child or that her child will have health problems, and she may repeatedly wash baby bottles and other baby supplies to alleviate this worry. In the long run, the behavior of repeatedly washing baby supplies is continuously strengthened and even generalized to washing other items and forcing her family to wash items. Encountering strong psychological stress or difficulties and setbacks that cannot be resolved will activate maladaptive thinking and behavior patterns.

In conclusion, in this study, a psychiatric diagnostic interview tool, the M.I.N.I., was used to evaluate the mental status of parturients. It was found that a variety of mental disorders occurred, including depressive disorders (mild) manic episode, generalized anxiety disorder, OCD, panic attacks, etc. The comorbidity rate was more than 50%, which seriously threatened the health of mothers and newborns. At present, the focus of postpartum health care is mainly on postpartum depression, while other types of postpartum mental disorders are rarely mentioned. For the mental and physical health of mothers and infants, it is very important to popularize science and education on a variety of mental disorders. More attention and support should be given to groups at high risk of postpartum mental disorders, such as individuals with a history of mental illness, pregnancy complications, partner violence, and no physical exercise, and early intervention should be carried out to prevent the occurrence and development of postpartum mental disorders. The results of this study showed that a high level of psychological stress may have a certain correlation with the occurrence of postpartum depression, anxiety disorders and OCD. It is necessary to evaluate the level of psychological stress related to childbirth and carry out early interventions. In our further work, we plan to incorporate IES-R evaluation into postpartum routine follow-up on 42 ± 7 days after delivery. Puerpera with IES-R score > 9 should be referred to our department of psychological medicine for systematic evaluation and necessary diagnosis and treatment. We hope our study will contribute to more comprehensive maternal mental and psychological health care.

### 4.4 Limitation

Regretfully, because of ethical limitations, the research could not be designed as a cohort study. What’s more, the stress levels were assessed within 1 month after delivery and some possible confounders, such as medications used after the delivery and history of psychological disorders were not be collected. In our further study, the stress levels during pregnancy, regular follow-up after childbirth and key details infected result should be brought into a more accurate evaluation.

## Data availability statement

The raw data supporting the conclusions of this article will be made available by the authors, without undue reservation.

## Ethics statement

The studies involving humans were approved by the Ethics Review Committee of Peking Union Medical College Hospital. The studies were conducted in accordance with the local legislation and institutional requirements. The participants provided their written informed consent to participate in this study.

## Author contributions

RS: Writing – original draft, Visualization. MZ: Data curation, Formal analysis, Investigation, Writing – original draft. LM: Resources, Writing – review & editing. YD: Writing – review & editing. JW: Funding acquisition, Resources, Supervision, Writing – review & editing.
